# Clinical trials in Charcot-Marie-Tooth disorders: a retrospective and preclinical assessment

**DOI:** 10.3389/fneur.2023.1251885

**Published:** 2023-09-22

**Authors:** Malavika A. Nair, Zhiyv Niu, Nicholas N. Madigan, Alexander Y. Shin, Jeffrey S. Brault, Nathan P. Staff, Christopher J. Klein

**Affiliations:** ^1^Department of Graduate Education, Alix School of Medicine, Rochester, MN, United States; ^2^Department of Laboratory Medicine and Pathology, Rochester, MN, United States; ^3^Department of Clinical Genomics, Rochester, MN, United States; ^4^Department of Neurology, Rochester, MN, United States; ^5^Division of Hand Surgery, Department of Orthopaedic, Rochester, MN, United States; ^6^Department of Physical Medicine and Rehabilitation Medicine, Mayo Clinic, Rochester, MN, United States

**Keywords:** CMT, clinical trials, neuropathy, gene therapy, CMT1A

## Abstract

**Objective:**

This study aimed to evaluate the progression of clinical and preclinical trials in Charcot-Marie-Tooth (CMT) disorders.

**Background:**

CMT has historically been managed symptomatically and with genetic counseling. The evolution of molecular and pathologic understanding holds a therapeutic promise in gene-targeted therapies.

**Methods:**

ClinicalTrials.gov from December 1999 to June 2022 was data extracted for CMT with preclinical animal gene therapy trials also reviewed by PubMed search.

**Results:**

The number of active trials was 1 in 1999 and 286 in 2022. Academic settings accounted for 91% and pharmaceutical companies 9%. Of the pharmaceutical and academic trials, 38% and 28%, respectively, were controlled, randomized, and double-blinded. Thirty-two countries participated: the United States accounted for 26% (75/286). In total, 86% of the trials were classified as therapeutic: 50% procedural (21% wrist/elbow surgery; 22% shock wave and hydrodissection therapy), 23% investigational drugs, 15% devices, and 11% physical therapy. Sixty-seven therapeutic trials (49%) were designated phases 1–2 and 51% phases 3–4. The remaining 14% represent non-therapeutic trials: diagnostic testing (3%), functional outcomes (4%), natural history (4%), and standard of care (3%). One-hundred and three (36%) resulted in publications. Phase I human pharmaceutical trials are focusing on the safety of small molecule therapies (*n* = 8) and AAV and non-viral gene therapy (*n* = 3). Preclinical animal gene therapy studies include 11 different CMT forms including viral, CRISPR-Cas9, and nanoparticle delivery.

**Conclusion:**

Current CMT trials are exploring procedural and molecular therapeutic options with substantial participation of the pharmaceutical industry worldwide. Emerging drug therapies directed at molecular pathogenesis are being advanced in human clinical trials; however, the majority remain within animal investigations.

## Introduction

Charcot-Marie-Tooth (CMT), also known as hereditary motor and sensory neuropathy (HMSN), refers to a group of inherited neuropathies of divergent genetic etiologies that affect motor greater than sensory peripheral nerves, typically without autonomic nervous system involvement (1). Collectively, CMT is globally the most common inherited neuropathy with PMP22 duplications (CMT1A) and its reciprocal deletion HNPP (hereditary neuropathy with pressure palsies) common. Specifically, PMP22 deletions are reported in 1:1698 newborns (2), and when considering the reciprocal duplication, a much higher overall rate must exist for PMP22 mutations. Clinical features of CMT typically include childhood or adolescence onset, gradual progressive declines, length-dependent motor deficits, stocking-glove sensory loss (large fiber sensory predominant), and foot and ankle deformities (pes cavus, hammertoes, and pes planus). Accompanying motor deficits include muscular atrophy in the legs, decreased deep tendon reflexes, and balance difficulties leading to frequent tripping and ankle sprains. Quality of life and disability measures are comparable to stroke patients when upper extremity hand dysfunction is included in the disability assessments (3). Exome sequencing has allowed for an understanding of the range of genotype–phenotype correlations, including the overlap of CMT with previously considered discordant disorders such as hereditary motor neuropathy (HMN) and hereditary sensory and autonomic neuropathies (HSAN) (4). When considering this overlap, there are well over 100 genes responsible for CMT phenotypes (1).

Historically, interventional therapies for CMT have been limited to supportive measures including genetic council, orthopedic support, and surgical interventions of the feet and ankles (5). Recently, drug-based genetic therapies have begun to emerge in other neuromuscular disorders including spinal muscular atrophy (6), transthyretin amyloidosis (7, 8), and adrenal leukodystrophy (9). These interventions have translated into meaningful improved morbidity and mortality with relative safety now approved by the FDA. Gene editing strategies include the application of targeted antisense oligonucleotide therapies, pre-RNA splicing therapy, gene replacement therapy by adeno-associated virus (AAV) viral vectors, clustered regularly interspaced short palindromic repeats (CRISPR) with CRISPR-associated protein 9 (Cas9) therapy, and lentivirus (LV) hematopoietic stem cell transplant. The success in the treatment of these previously fatal disorders has created optimism for the development of similar approaches in CMT patients.

In this study, we aimed to retrospectively assess the progression of clinical trials in CMT and review current laboratory strategies anticipating future therapeutic options.

## Methods

ClinicalTrials.gov was reviewed from December 1999 to June 2022 for CMT or HMSN. This included trials that contained CMT or HMSN as a MeSH term (keywords that are manually assigned by librarians at the National Library of Medicine) or included CMT and HMSN in the research links provided by the National Library of Medicine. Trials that were categorized as withdrawn, suspended, or terminated were excluded. The remaining trials were categorized based on various parameters, including intervention type (e.g., procedural, drug, device, and functional outcomes), study design, whether the trial occurred in an academic or pharmaceutical setting, trial status (recruiting, active, and completed), and the country where the trial was conducted. Each trial was further researched to determine whether any associated publications existed. Publications were verified by comparing titles, trial dates, and listed authors.

For the up-to-date review of animal studies, a PubMed search was performed for studies over the past 5 years. The queried search included the key terms “gene therapy”, “animal studies” and “Charcot-Marie-Tooth” or “Hereditary Motor and Sensory Neuropathy”. Identified manuscripts that were reviewed articles were included and the original manuscripts reviewed as related specifically to gene therapy.

## Results

### Human clinical-trial review

**Figure 1:**
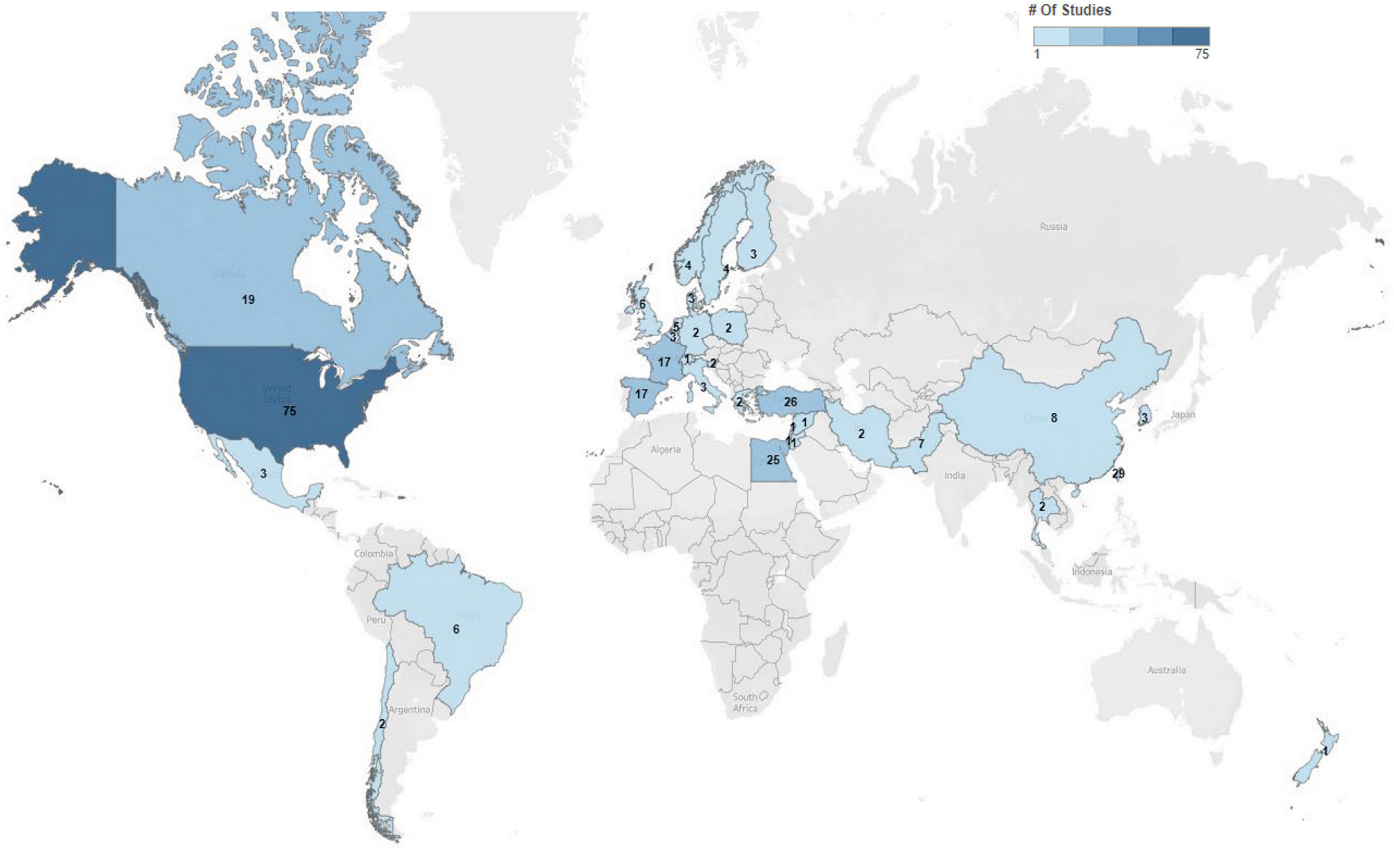
Charcot-Marie-Tooth trials by geographic location. Distribution and numbers of trials by geographic locations within clinicaltrials.gov 2022. Seen is the extent of trial across the world most commonly in the United States.

Five hundred eighty-nine trials were identified in clinicaltrials.gov, of which 286 of the current trials met the inclusion criteria, with details summarized along with available links to publications in the Supplementary Table 1. The largest number of trials occurred in the United States (26%), followed by Taiwan (10%) and Turkey (9%) (Figure 1). These trials were primarily conducted in academic settings (91%; 261/286), with the remainder conducted in pharmaceutical settings (9%; 25/286). In total, 29% (82/286) were randomized, double-blinded, and controlled, making up 28% (73/262) of academic trials and 38% (9/24) of pharmaceutical trials.

**Figure 2:**
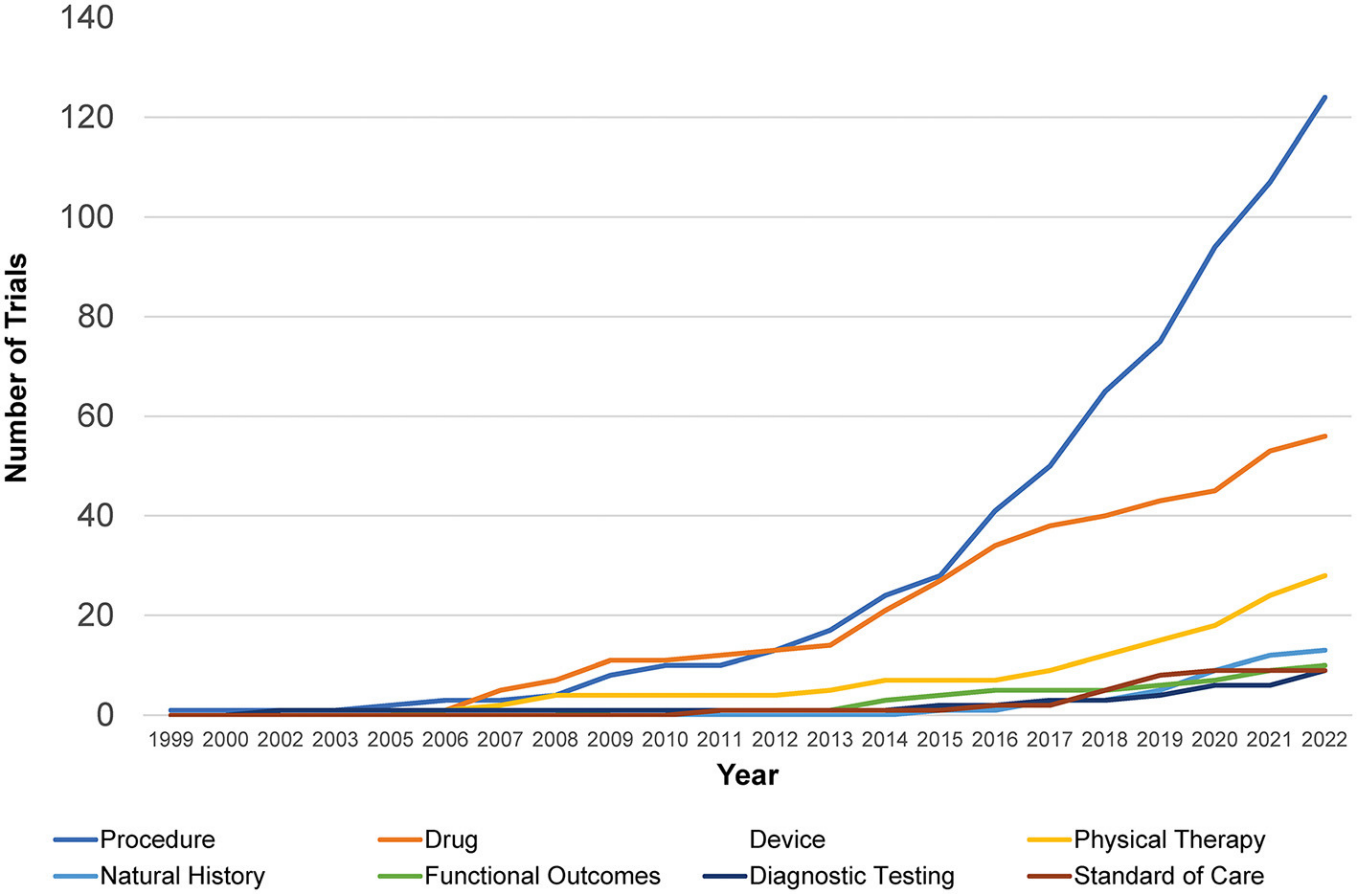
Retrospective Charcot-Marie-Tooth trials by intervention type. Shown is the distribution of different interventional trials within clinicaltrials.gov over time from 1999 to 2022 with an increase in procedural and drug studies seen most rapidly.

**Figure 3:**
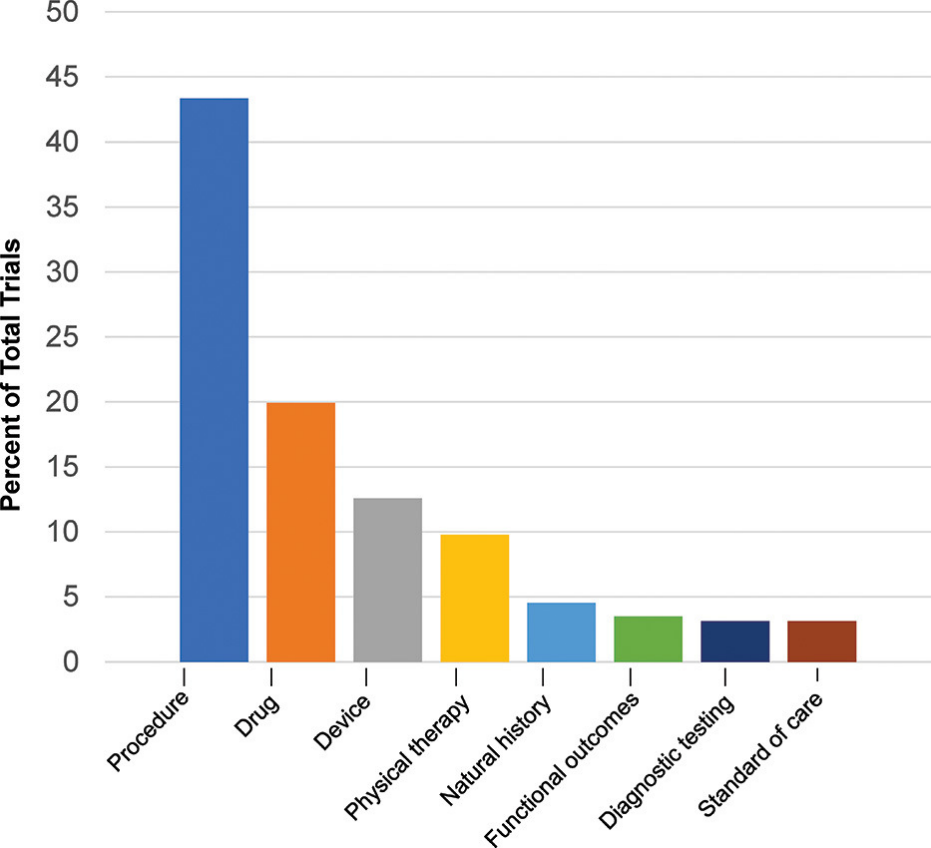
Current Charcot-Marie-Tooth trials by type. Current trials in clinicaltrials.gov 2022 show distribution of interventional and non-interventional trials.

The cumulative number of trials increased from 1 in 1999 to 41 in 2012 and 286 by 2022, with the most growth occurring in the last two decades (Figure 2). In total, 86% (245/286) of the trials were therapeutic testing of active interventions versus 14% non-therapeutic (diagnostic testing, functional outcomes, natural history, and standard of care). Most current therapeutic trials (50%; 124/245) were procedural interventions (Figure 3). The most frequently studied procedure was carpal/cubital tunnel release surgery (21%; 26/124), followed by extracorporeal shock wave therapy (11%; 14/125) and nerve hydrodissection therapy (11%; 14/124).

After procedural interventions, drug interventions were the most studied (20%; 57/286), followed by device interventions (13%; 36/286), physical therapy interventions (10%; 28/286), and trials investigating the natural history of disease (5%; 13/286). Of the therapeutic trials, 67 were designated with a specific phase. Trials in early phases 1 and 2 (49%; 33/67) and late phases 3 and 4 (51%; 34/67) were equally split.

Cumulative trials across all types of interventions have increased in the last decade from 40 to 286, rising noticeably from 34% in 2015 to 51% in 2022. Prior to then, procedural trials and drug trials had competed for the highest prevalence, with procedural trials finally taking the lead in 2013. At lower rates, trials investigating natural history of disease, diagnostic testing, standard of care, and functional outcomes have been comparable to each other over the past two decades. They have increased from 2.5 to 4% in the last decade, with natural history investigations taking a slight lead over the others in 2020.

From all trials, 36% (103/286) resulted in a publication; 35% (100/286) are still active or recruiting and, therefore, have not made it to the publication stage. Of the remaining, 28% (79/286) represent completed trials that have not been published. Only three trials have had results posted to ClinicalTrials.gov but with no corresponding publication. More recently, trials have started to investigate small molecule therapies (3%; 8/256), focusing specifically on the safety of utilizing these therapies. Additionally, trials investigating the use of AAV/non-viral gene therapy (1%; 3/286) in treating neuropathy have begun to emerge. Two additional trials from June of 2022 forward posted to ClinicalTrials.gov in 2023 not included in our 286 are a non-randomized trial titled “Research of SORD-CMT Natural History and Epalrestat Treatment” (NCT05777226). This study investigates the safety and efficacy of epalrestat, an aldose reductase inhibitor, in SORD-CMT2 patients and second to “The Safety and Tolerability of CLZ-2002 in Patients with Charcot-Marie Tooth Disease” (NCT05947578). CLZ-2002 is a tonsillar mesenchymal stem cell that is a neuronal regeneration-promoting cell (NRPC) delivered by intramuscular injection.

### Promising gene therapies in preclinical animal studies

From the PubMed search of animal gene therapy studies in CMT, 27 articles were most recently identified in July 2023 (10), with the most relevant studies with references listed (Table 1). Targeted gene therapy approaches include utilization of viral delivery by LV (*n* = 4), AAV (*n* = 9), CRISPR-Cas9 (*n* = 2), squalene nanoparticles (*n* = 1), and antisense oligonucleotides (*n* = 1). Each has shown early success including in phase 1 animal trials for both efficacy and safety.

**Table 1:** Preclinical animal and laboratory studies.

**Route of delivery**	**CMT**	**Delivery**	**Target**	**Stage**	**Mechanism result**
Intrathecal Intravenous	CMT2A	AAV8	SARM1	*In vivo*	Reduced axonal degeneration (11)
	CMT2D	AAV9	GARS	*In vivo*	Delayed axonal degeneration (12)
	CMT2E	CRISPR/Cas9	NEFL	*In vivo*	Phenotypic rescue (13)
	CMT2S	AAV9	IGHMBP2	Phase 1 clinical trial	Phenotypic rescue (14)
Intrathecal Intra-nerve Intravenous	CMT1X	AAV9	MPZ, GJB1	*In vivo*	Phenotypic rescue (15)
		LV	MPZ, GJB1	*In vivo*	Phenotypic improvement (16)
	CMT4C	LV	SH3TC2	*In vivo*	Incomplete rescue (17)
	CMT4J	AAV9	CBA, FIG4	*In vivo*	Incomplete rescue (18)
	CMT1B	LV	MANF	*In vivo*	Protective effect (19)
	CMT1A	AAV9	PMP22, shRNA	*In vivo*	Improved myelination (20)
			PMP22, miR	*In vivo*	Phenotypic rescue (21)
		LV	PMP22, shRNA	*In vivo*	Rescue of myelination (22)
		Squalene NP	PMP22, siRNA	*In vivo*	Functional recovery (23)
		CRISPR/Cas9	PMP22	*In vivo*	Myelination rescue (24)
		ASO	PMP22	*In vivo*	Phenotypic improvement (25)
Intra-muscular	CMT1A	AAV1	NT-3	Phase 1 clinical trial	Histologic functional improvement (26)
	CMT1X	AAV1	NT-3	*In vivo*	Phenotypic improvement (27)
	CMT2D	AAV1	NT-3	*In vivo*	Phenotypic improvement (28)

AAV, adeno-associated virus; LV, lentivirus; NP, nanoparticles; SARM1, sterile alpha and TIR motif containing 1; miR, microRNA; IGHMBP2, immunoglobulin mu DNA binding protein 2; MPZ, myelin protein zero; GJB1, gap junction beta 1; SH3TC2, SH3 domain and tetratricopeptide repeats 2; CBA, chicken β-actin; MANF, Mesencephalic astrocyte-derived neurotrophic factor; shRNA, small hairpin RNA; siRNA, short interfering RNA; NT-3, neurotrophin-3; HPHGF, human paracrine hepatocyte growth factor.

Articles not directed toward gene therapy but with animal benefits identified include utilization of sephin1/IFB-088/icerguastat in CMT1A and CMT1B, a non-specific unfolded protein response modulator (29); curcumin-cyclodextrin/cellulose nanocrystals antioxidant therapy in CMT1A (30); histone deacetylase 6 (HDAC6) inhibitor, SW-100, in neurodegeneration protection in CMT2A (31, 32); and exogenous pyruvate, a glycolytic product used in trembler^J^ PMP22 model, for distal axon energy supplement (33).

## Discussion

The results of this retrospective study of clinical trials demonstrate an increase in interventional research in the field of CMT over the past two decades, with identifiable evidence of progress toward more active interventions, and progress in disease-specific mechanism including gene therapy with additional early promise among animal studies. Participation from both academic and pharmaceutical companies is noted with trials occurring worldwide. Most recent trials are centered on identifying therapeutic approaches for CMT. The number of procedural therapies outweighs other therapeutic trials currently. Types of procedural trials included testing drugs and comparing imaging modalities, with many focused on investigating the outcomes of surgical procedures. Specifically, surgical procedures involving decompression of the carpal and ulnar tunnels were frequently studied procedures, with meaningful improvements in patient care having been published (34, 35). CMT1A patients have been shown to have meaningful improvement when affected by carpal tunnel syndrome and treated by surgical flexor retinaculum unroofing. Decompression surgery in these cases has been used to restore sensory and motor function in CMT patients and has decreased the recurrent symptoms in critical hand function, reducing disability. Patients with high carpal tunnel questionnaire scores and activity-induced symptoms are noted to benefit the most.

Extracorporeal shock wave therapy (ESWT) has also been investigated as a potential new therapy for other musculoskeletal disorders within different forms of peripheral neuropathies (36, 37). ESWT utilizes the transcutaneous application of acoustic waves theorized to suppress inflammatory reactions and promote neurogenesis and angiogenesis surrounding damaged and degenerative tissues including within tendons (38). It has been demonstrated to treat carpal tunnel syndrome by blinded sham control prospective study in non-CMT patients and has been described to encourage proliferation of Schwann cells and axonal regeneration during nerve repair (22). Although ClinicalTrials.gov lists ESWT with CMT, there are no publications and outcomes reported to date and will be needed before the acceptance of this approach with separate investigation likely needed in different CMT forms, given the range of pathogenic mechanisms.

The increase in the number of procedural trials represents a shift toward therapies that improve patient conditions beyond supportive care. In CMT patients, meaningful benefits of ankle foot surgery by tibialis posterior transfer and extensor hallucis longus transfer with calcaneal osteotomy for patients with marked cavovarus deformity (high arches and inward turning of the ankle) are found, in whom conservative orthosis and physical therapy interventions have failed (5, 39, 40). While this demonstrates progress in the procedural field, it also suggests a greater need for biological therapeutic engagement from academic and pharmaceutical participants, which is likely forthcoming based on our review of promising approaches currently being attempted in animals. Our review suggests <5% of human trials deal with AAV or small molecular therapies. However, the emergence of molecular approaches to human CMT therapies will be in accordance with safety and efficacy of emerging laboratory animal studies summarized with encouraging results including phase 1 animal trials underway (Table 1) (10). The described ongoing animal and laboratory-based studies emphasize the switch in focus to drug and gene therapies for CMT.

Specific trials in this review include a focus on the use of viral vectors in gene therapy and non-gene therapy by combinatorial drug therapy utilizing agent PXT3003. The PXT3003 includes three unlikely individual components, namely baclofen, naltrexone, and sorbitol. Baclofen is a GABA-B receptor agonist that is currently used to treat muscle spasticity; naltrexone is an opioid receptor antagonist that is used in the management of alcohol addiction; d-sorbitol is a muscarinic receptor antagonist used in the treatment of intestinal disorders. The combination of these drugs together has been shown to downregulate the expression of PMP22 mRNA and improve myelination (41). This novel therapy showed recent success in a phase 3 trial (NCT02579759), which confirmed its safety and tolerability, though it may not be widely accepted by the physicians and scientists in the CMT community. Ultimately, patients and physicians will consider cost vs. efficacy, and there may be a theoretical concern of potential long-term interference with central nervous system PMP22 expression (42). Regardless, the trial showed that patients in the group treated with high-dose PXT3003 showed improvements beyond stabilization without significant short-term side effects (43). It currently has been provided fast-track designation by the US Food and Drug Administration for CMT1A.

The limitations of our study are important to acknowledge as this study focused only on trials that were posted on ClinicalTrials.gov, not necessarily completed, so there may be a lack of reported research results. Nevertheless, we think the results do indicate the direction where the field is headed and therefore are meaningful. Additionally, geographic data may be biased as trials conducted in the United States may be more likely to be reported on ClinicalTrials.gov, leading to an underrepresentation of trials conducted in other countries. Furthermore, what is portrayed as an increase in the number of trials across all types of interventions could simply be researchers reporting their trials on ClinicalTrials.gov more consistently as the utilization of online databases has increased over the years.

Preclinical trials which are integral for gene therapy from animal models appear to be robust in this area. Viral and non-viral gene therapies in both rodent and non-human-primate models show exciting promise for clinical applications in CMT (10). Recent studies show that the majority of CMT gene therapy trials are in the *in vivo* phase and utilize adeno-associated virus and lentivirus as vectors. These studies have contributed significantly to the field of CMT and provide evidence of new pathways for therapy; they are important and predicted to be the major approach in approaching a cure in the diverse CMT disorders.

## Conclusion

This study provides evidence in CMT of increased investigational trials of both procedural and pharmacologic studies with increased involvement of both pharmaceutical and academic centers worldwide. The infrastructure created by these human clinical trials combined with the preclinical animal models of gene therapies will facilitate translational advancement in this relatively common group of disorders.

## Data availability statement

The original contributions presented in the study are included in the article/Supplementary material, further inquiries can be directed to the corresponding author.

## Ethics statement

Ethical review and approval was not required for the study on human participants in accordance with the local legislation and institutional requirements. Written informed consent from the patients/participants or patients/participants' legal guardian/next of kin was not required to participate in this study in accordance with the national legislation and the institutional requirements.

## Author contributions

MN and CK drafted the manuscript, performed data extraction, critical assessment of the data, and oversight of the study. ZN, NM, AS, JB, and NS critically reviewed the data providing critical edits and final approval. All authors contributed to the article and approved the submitted version.
